# Fungicidal versus fungistatic therapy of invasive *Candida* infection in non-neutropenic adults: a meta-analysis

**DOI:** 10.1080/21501203.2017.1421592

**Published:** 2018-01-09

**Authors:** Anand Kumar, Ryan Zarychanski, Amarnath Pisipati, Aseem Kumar, Shravan Kethireddy, Eric J. Bow

**Affiliations:** a Section of Critical Care Medicine, University of Manitoba, Winnipeg, Canada; b Department of Medical Microbiology and Infectious Diseases, University of Manitoba, Winnipeg, Canada; c Department of Pharmacology/Therapeutics, University of Manitoba, Winnipeg, Canada; d Department of Medical Oncology and Haematology, CancerCare, Winnipeg, Canada; e Department of Chemistry and Biochemistry, Laurentian University, Sudbury, Canada

**Keywords:** Invasive infection, candida, fungicidal, fungistatic, echinocandin, amphotericin B, triazole, therapy

## Abstract

The purpose of this study was to determine whether fungicidal versus fungistatic pharmacotherapy of invasive candidiasis/candidemia yields superior outcomes.

Data sources included MEDLINE (1966–June 2017), EMBASE (1980–June 2017), PubMed (1966–June 2017), Global Health-Ovid (inception to June 2017), LILACS Virtual Health Library (inception to June 2017) and the Cochrane Central Register of Controlled Trials (to 2nd quarter 2017). The ClinicalTrial.gov database, the SCOPUS database, SIGLE (System for Information on Grey Literature) and Google Scholar were also utilised to search for relevant studies.

Randomised studies of any pharmacotherapy of invasive candidiasis including candidemia using a fungicidal (amphotericin B or echinocandin compound) versus a fungistatic (triazole) compound in adolescent or adult non-neutropenic patients. Eight studies met the inclusion criteria.

Pooled odds ratios demonstrated an advantage of fungicidal therapy with respect to early therapeutic success (OR 1.61, 95% CI 1.27–2.03, *p* < 0.0001, *I*^2^ = 0%) and persistence or recurrence of infection (OR 0.51, 95% CI 0.35–0.74, *p* = 0.0005, *I*^2^ = 0%) but no advantage for late survival (OR 0.97, 95% CI 0.77–1.21, *p* = 0.77, *I*^2^ = 0%).

Fungicidal therapy of invasive candidiasis and candidemia is associated with a higher probability of early therapeutic success and decreased probability of persistent or recurrent infection. However, there is no improvement in survival.

As the severity and chronicity of illness of hospitalised patients has risen over the last several decades, the importance of *Candida* species as invasive pathogens has increased (Guinea 2014; Kullberg and Arendrup 2015). Epidemiological data show that *Candida* is 4th most common pathogen among nosocomial bloodstream infections although population-based studies suggest an overall ranking of 7th–10th place overall (Wisplinghoff et al. 2004; Kullberg and Arendrup 2015). Despite the relatively modest frequency of cases relative to some other pathogens, associated mortality can approach 50% (Leroy et al. 2009). In the past, most invasive *Candida* infections were seen in severely immunosuppressed patients (particularly in association with cytotoxic chemotherapy-induced neutropenia as well as with prolonged steroid use and solid organ transplants). However, with the increase in prevalence of chronic illness, non-immunosuppressed patients constitute an increasing portion of those suffering from invasive *Candida*. Contemporary risk factors for invasive *Candida* infection in these patients include the presence of a central venous catheter, use of total parenteral nutrition or prolonged broad-spectrum antibiotic therapy, recent surgical procedures, haemodialysis and high severity of illness (Yapar 2014; Kullberg and Arendrup 2015).

Until the introduction of triazoles in the mid-1980s, the only effective therapy for invasive candidiasis and candidemia was amphotericin B deoxycholate. Although highly fungicidal, nephrotoxicity limited its use to serious infections. Triazoles, unlike amphotericin B and the more recently released echinocandin compounds, are fungistatic for *Candida* species (Zhanel et al. 2001). Owing to the benign toxicity profile of triazoles, a major shift towards the use of this class of antifungal agents occurred following their introduction in the mid-1980s (Grasela et al. 1994). In the 15 years, several echinocandins have been introduced to clinical practice. Echinocandins, like amphotericin B, are highly fungicidal antifungal agents, but have substantially fewer side effects and minimal nephrotoxicity (Lass-Flörl et al. 2008).

For serious invasive *Candida* infections (particularly those associated with haemodynamic instability) where speed of clearance of the organism may potentially be a factor in outcome (Kumar 2014), the fungistatic activity of triazoles may represent a distinct disadvantage. Whether the use of triazoles for invasive *Candida* infections is associated with inferior clinical outcomes compared to fungicidal agents is uncertain. Given the increasing frequency of invasive *Candida* infections in among non-neutropenic patients, a meta-analysis of all adult randomised studies in which the clinical outcome of therapy with a triazole was compared to therapy with either amphotericin B or an echinocandin in such patients was performed.

## Methods

### Study selection

The primary purpose of this study was to determine assess the comparative efficacy and safety of a fungistatic regimen (triazole therapy) in comparison to a fungicidal therapy (amphotericin B or echinocandin) in adolescent and adult non-neutropenic patients with invasive *Candida* infection. Studies were included if they met the following criteria: therapy with a triazole agent was compared to either therapy with amphotericin B or an echinocandin; the study population primarily (>90%) comprised non-neutropenic patients or non-neutropenic patients could be extracted from the data set; patients had a documented invasive *Candida* infection (candidemia or alternately a positive biopsy or culture from a normally sterile site); treatment groups were randomly assigned; and enrolled patients were ≥12 years of age. Studies examining superficial mycoses (including cystitis, mucositis and oesophageal candidiasis) in the absence of invasive disease or focused on neutropenic patients were excluded. There was no restriction on the use of other antimicrobial agents (including supplemental antifungal agents such as flucytosine). Since in vitro studies have suggested antimicrobial indifference between amphotericin B and fluconazole (Ghannoum et al. 1995; Sugar 1995), the addition of this triazole to amphotericin B was also acceptable in the fungicidal therapy group.

All studies had to provide sufficient data on fungistatic versus fungicidal antimicrobial therapy to calculate an odds ratio (OR) between treatment groups. Confirmed sensitivity of the isolated organism was not required since sensitivity testing of *Candida* isolates was not available through much of the study period. Frequency of therapeutic success and survival were the primary outcome measures. Microbiological persistence of infection and evidence of nephrotoxicity were secondary endpoints.

### Data sources

Relevant studies for our meta-analysis were identified from MEDLINE (1966–June 2017), EMBASE (1980–June 2017), PubMed (1966–June 2017), Global Health-Ovid (inception to June 2017), LILACS Virtual Health Library (inception to June 2017) and the Cochrane Central Register of Controlled Trials (to 2nd quarter 2017). The ClinicalTrial.gov database, the SCOPUS database, SIGLE (System for Information on Grey Literature) and Google Scholar were also utilised to search for relevant studies. Finally, to identify other ongoing or planned trials, we searched the World Health Organization’s International Clinical Trials Registry Platform for relevant trial registrations. The search was conducted in part by information specialists of the University of Manitoba. Search terms included “candidemia”, ”candidiasis”, “fungal infections” or ”fungemia” cross-indexed against “amphotericin B”, ”echinocandin”, “antifungals”, “triazoles”, “micafungin”, “anidulafungin”, “caspofungin”, “fluconazole”, “itraconazole”, “voriconazole”, and “posaconazole” and restricted to “randomized control trial” or “clinical trial”. References from relevant articles and recent published reviews and recent meta-analyses on related topics were also reviewed to identify additional relevant studies. Meeting abstracts of the Interscience Conference on Antimicrobial Agents and Chemotherapy, the American Society for Microbiology, the Infectious Diseases Society of America, the Society for Healthcare Epidemiology of America and the Society of Critical Care Medicine were reviewed from 1996 to 2016. No language restrictions were applied.

### Study selection and data extraction

We used a two-stage process for study selection. First, two independent reviewers (AK, SK) screened all citations (title and, when available, abstract) for relevant eligible studies. Each report was classified as follows: include, exclude, unclear, or duplicate of another citation. The full text of all citations classified as “include” or “unclear” by either reviewer were retrieved for formal review. A pre-piloted standard data form outlining the inclusion and exclusion criteria was used to extract relevant data from the eligible articles independently by two reviewers (AK, SK). Final selection of studies was done by consensus.

The methodological quality of included studies was assessed by the two unblinded reviewers (RZ, AK) at the study level using the Jadad scale (value range 0–5) which considers factors including randomisation, blinding and participant withdrawals (Jadad et al. 1996) (Table 1). A score of 5 was considered “high” methodological quality. Allocation concealment was assessed using the method developed by Schultz and colleagues (Schulz et al. 1995) (Table 1). Information regarding methodological quality and potential risks of bias were used to guide sensitivity analyses and explore sources of heterogeneity.

**Table 1.** Methodological quality and potential risks of bias in the included randomised controlled trials.

			Jadad score^a^					
Study/year	RCT type	Sponsor	Total	Randomisation	Blinding	Attrition information	Allocation concealment	ITT analysis
Kujath et al. (1993)	Single centre	NR	2	1	0	1	Unclear	Unclear
Rex et al. (1994)	Multicentre	Roerig-Pfizer, NIH	3	2	0	1	Adequate	Yes^b^
Abele-Horn et al. (1996)	Single centre	NR	3	2	0	1	Unclear	Unclear
Anaissie et al. (1996)	Multicentre	Roerig-Pfizer	3	2	0	1	Adequate	Yes
Phillips et al. (1997)	Multicentre	Pfizer	2	1	0	1	Adequate	Yes^b^
Rex et al. (2003)	Multicentre	Pfizer, NIH	5	2	2	1	Adequate	Yes^b^
Kullberg et al. (2005)	Multicentre	Pfizer	3	2	0	1	Adequate	Yes^b^
Reboli et al. (2007)	Multicentre	Vicuron/Pfizer	4	2	1	1	Adequate	Yes^b^

^a^The Jadad scale assigns methodological quality score based on the reported methods and description of randomisation (0–2 points), blinding (0–2 points) and the reporting of participant withdrawals (0–1 point). Possible scores vary from 0 to 5, with a score of 5 indicating high methodological quality. ^b^Modified intention-to-treat analysis. RCT: randomised controlled trial; NR: not reported; ITT: intention to treat.

The following data was collected for each experimental group: frequency of successful therapy, definition of successful therapy, survival to hospital discharge, frequency of microbiological persistence or recurrence at the end of therapy, frequency and definition of renal toxicity/injury, drug dose, duration of primary antifungal therapy and use and duration of supplemental antifungal therapy (flucytosine or triazole with the primary agent). If required data were ambiguous or missing, the authors of the study were contacted for clarification or additional data. Incongruent elements were resolved by consensus. Since our interest was in non-neutropenic patients, where possible, neutropenic subjects were eliminated from the data set.

#### Data synthesis

We analysed discrete and continuous data using the Cochrane Review Manager (version 5.2.11). ORs and 95% confidence intervals (CIs) were calculated. We employed a random-effects model using inverse variance weights for all summary measures of effect. For dichotomous data, we expressed summary measures of effect across studies as OR with 95% CIs. An OR of >1 suggested a higher occurrence of the outcome (e.g. survival) among patients given fungicidal agent (i.e. amphotericin B or an echinocandin) compared to the group receiving fungistatic therapy (i.e. a triazole drug). A ratio <1 indicated a higher occurrence of the outcome of interest in the fungistatic therapy group.

We assessed evidence of statistical heterogeneity using the *I*^2^ statistic. This statistic can be interpreted as the proportion of total variation across studies attributable to heterogeneity (value range, 0–100%). We investigated potential sources of heterogeneity by conducting sensitivity analyses based on study characteristics. The data did not permit analyses based on the total dose of drug delivered. Despite the relatively small number of studies, a funnel plot of the log of the OR against the sample size was performed for the primary study endpoints to assess for potential publication bias.

## Search results

Of the 522 records initially identified, 396 were excluded after initial screening of the title and/or abstract: 151 were duplicate records; 285 were excluded for other reasons including being animal or in vitro investigations. The remaining 86 underwent evaluation of the full-text manuscript. Seventy-eight reports were found to be non-randomised, prospective studies or retrospective investigations (62 studies). The remaining 16 studies involved non-invasive disease (16 studies). Eight studies were found to meet entry criteria and were selected for inclusion in the meta-analysis (Kujath et al. 1993; Rex et al. 1994, 2003; Abele-Horn et al. 1996; Anaissie et al. 1996; Phillips et al. 1997; Kullberg et al. 2005; Reboli et al. 2007).

### Study characteristics

A total of 1335 eligible subjects were enrolled in the eight included trials (Kujath et al. 1993; Rex et al. 1994, 2003; Abele-Horn et al. 1996; Anaissie et al. 1996; Phillips et al. 1997; Kullberg et al. 2005; Reboli et al. 2007) (Table 2). All of the studies were published in peer-reviewed, English-language journals between 1993 and 2014. The total number of eligible subjects in the studies ranged from 40 to 370. Of the eight studies, half enrolled more than 100 eligible patients (Rex et al. 1994, 2003; Kullberg et al. 2005; Reboli et al. 2007) and 6 were multicentre (Rex et al. 1994, 2003; Anaissie et al. 1996; Phillips et al. 1997; Kullberg et al. 2005; Reboli et al. 2007). Although all studies were randomised, only two claimed blinding through the study (Rex et al. 2003; Reboli et al. 2007). In two cases, the study was blinded at initial randomisation but not during the entire course of the study (Rex et al. 1994; Kullberg et al. 2005) (Table 1).

**Table 2.** Study descriptions.

Study (year)	Age (years)	Documented candidemia required	Fungistatic therapy and daily dose^a^	Duration	Fungicidal therapy and daily dose^a^	Duration	Supplemental therapy	Definition of renal injury/dysfunction	Clinical success definition	Death at
Kujath et al. (1993)	≥18	No	Fluconazole 300 mg	9 ± 5 d SD	Amphotericin B deoxycholate 0.5 mg/kg	13 ± 8 d SD	Flucytosine 7500 mg/day with cidal group	Cr > 2.0 X baseline	Microbiological clearance at EOT	Hospital discharge
Rex et al. (1994)	≥13	Yes	Fluconazole 400 mg	14 days post-clinical resolution and last positive BC	Amphotericin B deoxycholate 0.5–0.6 mg/kg	14 days post-clinical resolution and last positive BC		Cr > 3.5 mg/dL	Clinical and microbiological resolution at EOT	15 weeks
Abele-Horn et al. (1996)	≥18	No	Fluconazole 200 mg	15 ± 9 days SD	Amphotericin B deoxycholate 0.5–0.75 mg/kg	15 ± 9 days SD	Flucytosine 7500 mg/day with cidal group	Unclear	Clinical and microbiological resolution at EOT	EOT
Anaissie et al. (1996)	≥13	No	Fluconazole 400 mg	Variable 9 days median	Amphotericin B deoxycholate 0.67 mg/kg	250 mg min total/9 days median		Unclear	Clinical and microbiological resolution at EOT	EOT
Phillips et al. (1997)	≥18	Yes	Fluconazole 400 mg	4–8 weeks (high end if metastatic)	Amphotericin B deoxycholate 0.6 mg/kg	2–5 weeks (high end if metastatic)		Cr > 1.5× baseline	Clinical and microbiological resolution at 7 days therapy	24 weeks
Rex et al. (2003)	≥13	Yes	Fluconazole 800 mg	14 days post-clinical resolution and last positive BC	Amphotericin B deoxycholate 0.7 mg/kg	14 days post-clinical resolution and last positive BC	Fluconazole 800 mg/day to EOT after 5–8 days amphotericin B	Cr > 3.5 mg/dL	Clinical and microbiological resolution at EOT	13 weeks
Kullberg et al. (2005)	≥12	Yes	Voriconazole 6 mg/kg	Minimum 14 days post-clinical resolution and last positive BC (8 weeks max)	Amphotericin B deoxycholate 0.7–1.0 mg/kg	Minimum 14 days post-clinical resolution and last positive BC (8 weeks max)	Fluconazole after 3–7 days for cidal therapy group	Cr > 2.0× baseline	Clinical resolution at last available study visit (EOT to 12 weeks post EOT)	14 weeks
Reboli et al. (2007)	≥16	No	Fluconazole 400 mg	14 days post-clinical resolution and last positive BC	Anidulafungin 100 mg	14 days post-clinical resolution and last positive BC	Oral fluconazole an option after 10 days IV therapy		Clinical and microbiological resolution at EOT	9 weeks

^a^After loading dose, EOT: end of therapy, BC: blood culture, max: maximum, IV: intravenous.

Three studies enrolled only adults (age 18 years or more) (Kujath et al. 1993; Abele-Horn et al. 1996; Phillips et al. 1997). The others enrolled adults and older children with the study by Kullberg et al. (2005) enrolling children as young as 12. Candidemia was the enrolment criterion in four of the trials (Rex et al. 1994, 2003; Phillips et al. 1997; Kullberg et al. 2005); the others allowed entry on the basis of invasive candidiasis without documented candidemia (Kujath et al. 1993; Abele-Horn et al. 1996; Anaissie et al. 1996; Reboli et al. 2007). Five of the studies specifically excluded neutropenic subjects (Rex et al. 1994, 2003; Abele-Horn et al. 1996; Phillips et al. 1997; Kullberg et al. 2005). In one study, invasive Candida cases associated with neutropenia (polymorphonuclear leucocyte count <1000/μL) were removed from the data set with only the remaining cases retained for analysis (Anaissie et al. 1996). In another study, only non-neutropenic (post-surgery) subjects appear to have been enrolled although an absence of neutropenia was not an explicit study requirement (Kujath et al. 1993). There were 2.5% patients with neutropenia in the remaining study (Reboli et al. 2007). This was the only study in which the fungicidal arm was represented by an echinocandin.

In seven trials, the fungistatic therapy utilised was fluconazole (Kujath et al. 1993; Rex et al. 1994, 2003; Abele-Horn et al. 1996; Anaissie et al. 1996; Phillips et al. 1997; Reboli et al. 2007); the remaining study utilised voriconazole (Kullberg et al. 2005). Seven of the eight studies used standard amphotericin B deoxycholate as the fungicidal therapy (Kujath et al. 1993; Rex et al. 1994, 2003; Abele-Horn et al. 1996; Anaissie et al. 1996; Phillips et al. 1997; Kullberg et al. 2005); the other study used the echinocandin, anidulafungin (Reboli et al. 2007). No trial utilised lipid-modified amphotericin B preparations. In two cases, amphotericin B deoxycholate was supplemented with flucytosine for a variable duration (Kujath et al. 1993; Abele-Horn et al. 1996). In one case, amphotericin B was supplemented by fluconazole at the same dose used in the fungistatic therapy arm (Rex et al. 2003). For the fungistatic arm, the dose of fluconazole ranged between 200 and 800 mg daily; most studies utilised a dose of 400 mg/day (Rex et al. 1994; Anaissie et al. 1996; Phillips et al. 1997; Reboli et al. 2007) (Table 2). Voriconazole was used at a dose of 6 mg/kg/day. For the fungicidal arm, amphotericin B was utilised at a final dose of 0.5–1.0 mg/kg/day with most trials using 0.6–0.7 mg/kg (Table 2); anidulafungin was studied at 100 mg/day (Reboli et al. 2007).

The duration of therapy varied widely (Table 2). In four cases, therapy in both arms was mandated to continue for at least 14 days following both clinical resolution and last positive pathogen culture (Rex et al. 1994, 2003; Kullberg et al. 2005; Reboli et al. 2007). In three studies, the duration of therapy in both groups was variable depending on the clinical response (Abele-Horn et al. 1996; Anaissie et al. 1996; Phillips et al. 1997). In one case, it was undefined and variable (Kujath et al. 1993).

The primary reported study outcome in all of the included studies was therapeutic success described as “clinical cure” or “successful” therapy. All studies also provided data on the secondary endpoints that included survival, microbiological persistence and renal injury. The definition of “successful” therapy or “clinical cure” included resolution of clinical signs and symptoms of infection along with initial microbiological clearance from blood and/or tissues in seven of eight cases (Rex et al. 1994, 2003; Abele-Horn et al. 1996; Anaissie et al. 1996; Phillips et al. 1997; Kullberg et al. 2005; Reboli et al. 2007). In one case, microbiological clearance without reference to clinical response was sufficient (Kujath et al. 1993). Microbial persistence or recurrence indicated persistence or recurrence of the same pathogen within the duration of the study observation period. The definition of renal injury was inconsistent. In most cases, this was defined by a specific elevation in serum creatinine or blood urea nitrogen but the amount was highly variable ranging from any significant elevation (Rex et al. 1994; Abele-Horn et al. 1996) to an increase to over 3.5 mg/dL (serum creatinine) (Rex et al. 2003) or doubling over baseline (Kujath et al. 1993; Kullberg et al. 2005) (Table 2).

Therapeutic success was generally determined at end of therapy (Kujath et al. 1993; Rex et al. 1994, 2003; Abele-Horn et al. 1996; Anaissie et al. 1996; Reboli et al. 2007) except in two cases. In one, it was defined at the end of 1 week of therapy (Phillips et al. 1997), and in the other it was defined at the last available study visit between end of therapy and 12 weeks following the end of therapy (Kullberg et al. 2005). The duration of follow-up used for assessment of other summary effects including rates death, microbiological persistence and drug toxicity was 9–24 weeks in five cases (Rex et al. 1994, 2003; Phillips et al. 1997; Kullberg et al. 2005; Reboli et al. 2007). In two studies, the assessment period was the duration of antifungal therapy (Abele-Horn et al. 1996; Anaissie et al. 1996), and in one, the entire period of hospitalisation (Kujath et al. 1993).

### Data synthesis

#### Successful therapy

**Figure 1.**
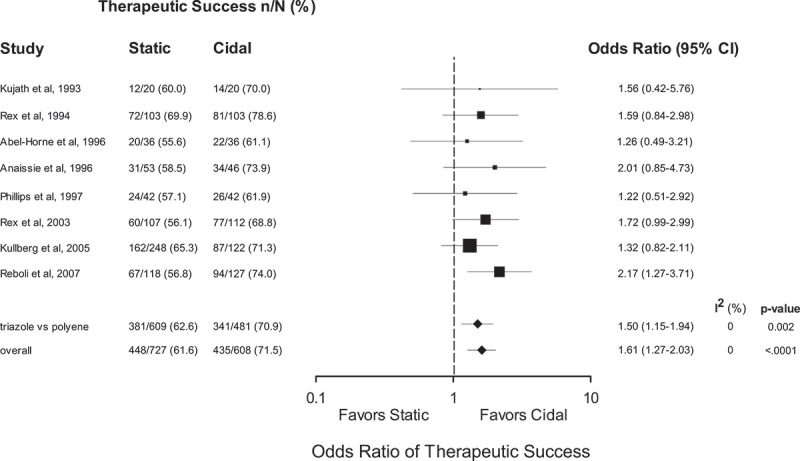
Therapeutic success at end of therapy for trials comparing fungicidal and fungistatic therapy of invasive candidiasis and/or candidemia. Odds ratios (ORs) are pooled using a random-effects model on a logarithmic scale. The size of the squares is proportional to the reciprocal of the variance of the studies. CI: confidence interval, n/N: frequency of finding.

Data from the eight included trials were pooled in two steps to generate a summary OR for successful therapy (Figure 1). First, all seven studies using amphotericin B in the fungicidal therapy arm were pooled (Kujath et al. 1993; Rex et al. 1994, 2003; Abele-Horn et al. 1996; Anaissie et al. 1996; Phillips et al. 1997; Kullberg et al. 2005). The pooled summary effect estimate was 1.50 (95% CI 1.15–1.94, *p* = 0.002, *I*^2^ = 0%), suggesting a highly consistent effect favouring fungicidal over fungistatic therapy with respect to success of therapy (Kujath et al. 1993; Rex et al. 1994, 2003; Abele-Horn et al. 1996; Anaissie et al. 1996; Phillips et al. 1997; Kullberg et al. 2005). This effect was strengthened (OR 1.61, 95% CI 1.27–2.03, *p* < 0.0001, *I*^2^ = 0%) with the addition of the Reboli study of anidulafungin (Reboli et al. 2007), suggesting the effect is not unique to amphotericin B. All individual studies favoured the fungicidal therapy arm including the two studies that included flucytosine as supplemental therapy.

#### Microbiological persistence of infection

**Figure 2.**
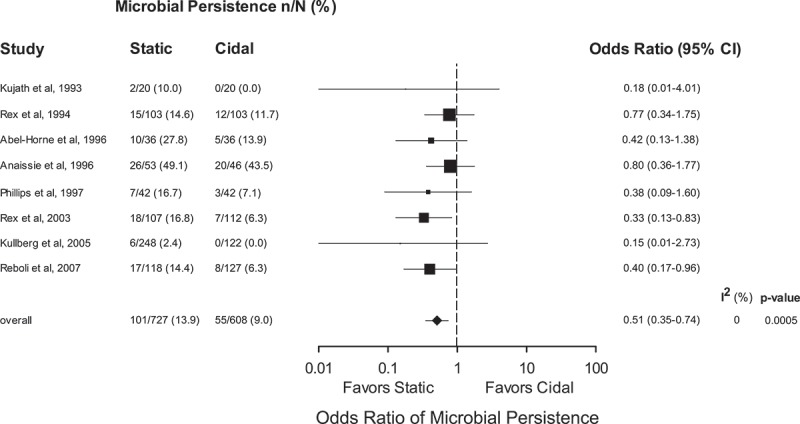
Microbial persistence following therapy for trials comparing fungicidal and fungistatic therapy of invasive candidiasis and/or candidemia. Odds ratios (ORs) are pooled using a random-effects model on a logarithmic scale. The size of the squares is proportional to the reciprocal of the variance of the studies. CI: confidence interval, n/N: frequency of finding.

Microbiological persistence was similarly consistent across all trials. In each case, individual study results fell in favour of the fungicidal therapy group (Figure 2). The pooled summary effect estimate (OR 0.51, 95% CI 0.35–0.74, *p* = 0.0005, *I*^2^ = 0%) suggests a marked disadvantage to fungistatic therapy with regard to microbiological persistence of *Candida* infection at the end of therapy.

#### Survival

**Figure 3.**
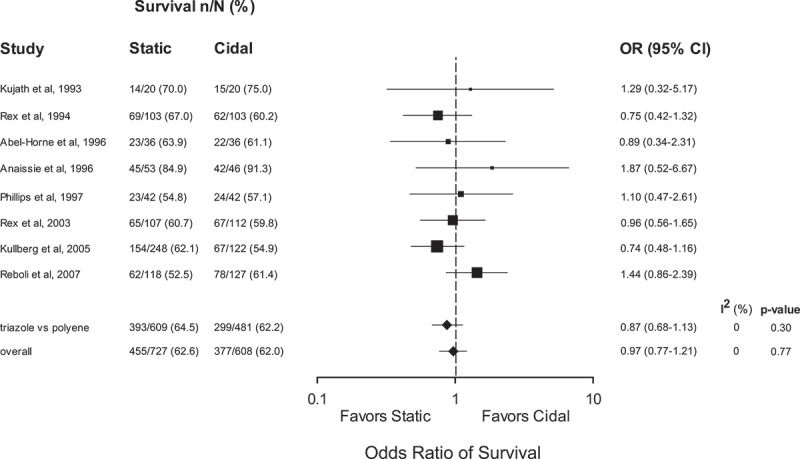
Survival to hospital discharge following therapy for trials comparing fungicidal and fungistatic therapy of invasive candidiasis and/or candidemia. Odds ratios (ORs) are pooled using a random-effects model on a logarithmic scale. The size of the squares is proportional to the reciprocal of the variance of the studies. CI: confidence interval, n/N: frequency of finding.

Although not a primary endpoint for any study, all studies provided information on survival. Data from the eight included trials were pooled in two steps to generate a summary effects estimate for survival (Figure 3). First, the seven studies using amphotericin B in the fungicidal therapy arm were pooled (Kujath et al. 1993; Rex et al. 1994, 2003; Abele-Horn et al. 1996; Anaissie et al. 1996; Phillips et al. 1997; Kullberg et al. 2005). The OR for these studies was 0.87 (95% CI 0.68–1.13, *p* = 0.30, *I*^2^ = 0%). With the addition of the final study (Reboli et al. 2007) using the echinocandin anidulafungin (individual OR 1.44, 95% CI 0.86–2.39), the pooled OR approaches unity (OR 0.97, 95% CI 0.77–1.21, *p* = 0.77, *I*^2^ = 0%).

#### Renal injury

**Figure 4.**
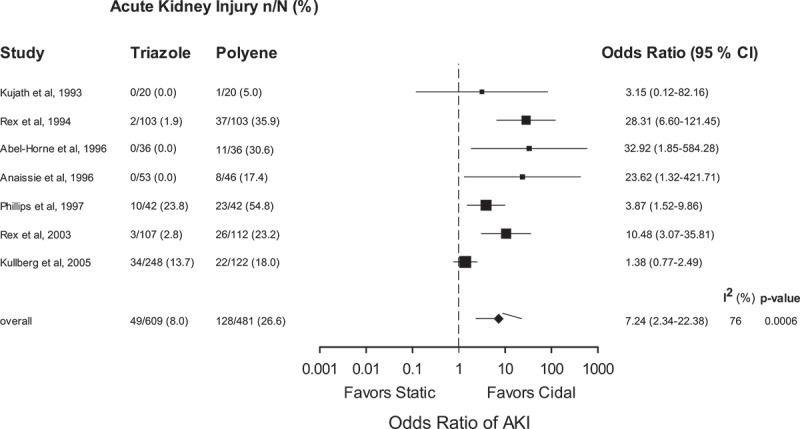
Renal injury during or following therapy for trials comparing fungicidal and fungistatic therapy of invasive candidiasis and/or candidemia. Only the seven studies including amphotericin B in the fungicidal arm are included. Odds ratios (ORs) are pooled using a random effects model on a logarithmic scale. The size of the squares is proportional to the reciprocal of the variance of the studies. CI: confidence interval, n/N: frequency of finding, AKI: acute kidney injury.

Because renal dysfunction is not a typical manifestation of the toxicity of echinocandins, this variable was examined only as it pertained to therapy with studies involving the use of amphotericin B (Kujath et al. 1993; Rex et al. 1994, 2003; Abele-Horn et al. 1996; Anaissie et al. 1996; Phillips et al. 1997; Kullberg et al. 2005). Amphotericin B therapy was associated with a higher degree of renal dysfunction in all individual studies with a pooled OR of 7.24 (95% CI 2.34–22.38, *p* = 0.0006, *I*^2^ = 77.6%) (Figure 4). While uniformly associated with increased renal injury, statistical heterogeneity (*I*^2^ value = 76%) for this variable was high, suggesting substantial variation in the frequency of nephrotoxicity. Heterogeneity of the pooled estimate may be due to: (a) variations in the risk of renal injury with fluconazole and voriconazole as the fungistatic drugs and/or (b) wide variation in criteria for diagnosis of renal injury.

The diagnostic criteria for renal injury varied from 1.5- to 2-fold increases of serum creatinine (Kujath et al. 1993; Phillips et al. 1997; Kullberg et al. 2005) from baseline in three studies and absolute increases in creatinine to over 3.5 mg/dL in two studies (Rex et al. 1994, 2003) (Table 2 for renal injury definitions). In the remaining trials, the definition of renal injury was not clear. When the two studies that used fluconazole as the fungistatic comparator and defined renal dysfunction as a rise in creatinine >1.5–2× baseline were assessed, *I*^2^ fell to 0% (Kujath et al. 1993; Phillips et al. 1997). In the two studies with fluconazole as the comparator and that defined renal injury as a rise in serum creatinine to >3.5 mg/dL, statistical heterogeneity was also substantially reduced (*I*^2^ = 6%) (Rex et al. 1994, 2003).

#### Publication bias

We minimised the potential for publication bias by conducting a thorough literature search that included searching grey literature and consulting with content experts. Funnel plots for therapeutic success and survival demonstrated no asymmetry to suggest publication bias. However, the inclusion of only eight trials in this analysis limits the reliability of this assessment.

## Discussion

A rapidly evolving body of research literature suggests that optimisation of antimicrobial therapy can improve outcome in severe, life-threatening infections (Kumar 2014). Approaches such as extended or continuous infusion of β-lactams, once-daily dosing of aminoglycosides and use of synergistic combinations of antibiotics share a common effect of increased pathogen clearance, i.e. augmented bactericidal activity. This principle has been shown most clearly in serious bacterial infections. In this meta-analysis, we have attempted to extend this observation to antimicrobial therapy of serious, invasive *Candida* infections in adolescent and adult non-neutropenic patients. Our analysis shows that fungicidal therapy with echinocandins and amphotericin B yields superior initial cure/successful therapy rates (Figure 1) and reduced microbial persistence (Figure 2) than fungistatic therapy with triazoles in invasive *Candida* infection. However, despite the difference in initial therapeutic success and microbial persistence, survival is ultimately similar in the two groups (Figure 3). One explanation for this may be that it is a consequence of the late intrinsic renal toxicity of amphotericin B (Figure 4) since renal injury is known to worsen outcome in critically ill patients (Fan et al. 2012). Unlike the pooled amphotericin B studies, the anidulafungin study (Reboli et al. 2007) demonstrated a modest trend to improved survival along with higher therapeutic success and lower microbiological persistence in the fungicidal therapy group. Overall, our analysis supports international recommendations that anti-*Candida* therapy with fungicidal activity but limited toxicity (i.e. echinocandins) should be preferred in seriously ill, adult non-neutropenic patients with invasive *Candida* infections (Bow et al. 2010; Cornely et al. 2012; Pappas et al. 2015).

The superiority of echinocandins compared to triazoles in terms of therapeutic success has been established for anidulafungin compared to fluconazole in one major randomised controlled trial (Reboli et al. 2007). It is also supported by recent retrospective analyses (Chiotos et al. 2016). However, the superior therapeutic success rate (despite the absence of an ultimate survival benefit) with amphotericin B in randomised trials is a novel observation (Figure 1). This finding is supported by a parallel analysis that shows that microbiological persistence of *Candida* is prolonged with triazole compared with amphotericin B therapy (Figure 2).

The reason for an absence of improved survival in the context of a higher therapeutic success rate with amphotericin B is uncertain. Based on the definitions in the included studies, a higher therapeutic success rate represents only early clinical improvement and/or early survival, usually at the end of therapy. The most plausible possibility for better therapeutic success frequency may be the more rapid clearance of pathogen that should be inherent with the use of a fungicidal drug (all other things being equal). The underlying hypothesis driving this analysis was that the microbial burden during severe infectious illness is based, in part, on an increasing microbial burden relative to less severe illness and that persistence of severe illness drives an increased risk of death (Kumar 2014). Accelerated pathogen clearance (as expected by definition with microbicidal therapy) in severe infections should presumably result in more rapid illness resolution and improved survival. This possibility is consistent with the observation of equivalent rates of clinical cure in randomised controlled trials of amphotericin B formulations versus echinocandins as treatment for invasive *Candida* infections (Mora-Duarte et al. 2002; Kuse et al. 2007)

The question of why an improvement in early therapeutic success fails to translate into improved survival, particularly with amphotericin B, is intriguing. Several possibilities exist each of which may have therapeutic implications. It is certainly possible that the advantage of the fungicidal activity of amphotericin B (which explains superior early therapeutic success rate) is offset by significant toxicity risks (which may lead to late organ injury-related complications and death). Nephrotoxicity associated with amphotericin B has been shown in several data sets to contribute to increases in mortality, longer hospitalisations and markedly increased hospital costs. Reports suggest 2.7- to 6-fold increase in the odds of death following the occurrence of amphotericin B-associated nephrotoxicity (Bates et al. 2001; Harbarth et al. 2002; Chen et al. 2006). Among the pooled studies included in our meta-analysis, the incidence of nephrotoxicity was approximately three times higher (26.6% vs. 8%) in the amphotericin B treatment groups when compared to triazoles (Figure 4).

If so, this would suggest that the ideal therapy for life-threatening invasive *Candida* infections and candidemia is a fungicidal therapy with low toxicity such as an echinocandin. In contrast to the results seen with amphotericin B therapy, the only study comparing an echinocandin (anidulafungin) to a triazole (fluconazole) demonstrated an improved early therapeutic success frequency (74.0% vs. 56.8%, OR 17.2 CI 5.5–29.0) with a modest trend towards improved late survival (31% vs. 23%, *p* = 0.14) in the therapy of invasive *Candida* infection (Reboli et al. 2007). However, when this option is not available or in resource-limited environments, a reasonable strategy may be a very short course of amphotericin B followed by completion of therapy using a fungistatic compound (similar to the approach used by Rex et al. 2003).

Evidence supporting superiority of microbicidal over microbiostatic therapy is limited to experimental animal studies and observational data. While clinical trials to directly test the presumption of the superiority of microbicidal activity for a broad range of infection severity are inconsistent, the case for microbicidal drug therapy in severe life-threatening infections appears stronger (Duke et al. 2002; Reboli et al. 2007; Prasad et al. 2012; Kumar 2014). Animal studies have shown that fungicidal therapy does result in accelerated tissue pathogen clearance in animal models of invasive candidiasis (Krishnan-Natesan et al. 2010). Although other meta-analyses or patient-level quantitative reviews of individual drug therapies for invasive candidiasis and/or candidemia (Kontoyiannis et al. 2001; Gafter-Gvili et al. 2008; Mills et al. 2009; Andes et al. 2012) have been published, none have attempted to exclude non-neutropenic patients or have focused on the issue of fungicidal versus fungistatic activity as a factor in outcome. Two meta-analyses failed to demonstrate a difference in survival between patients treated with amphotericin B and triazole therapy (Gafter-Gvili et al. 2008; Mills et al. 2009) but one did suggest an increased *Candida* persistence with triazole compared to amphotericin B therapy (Gafter-Gvili et al. 2008). Several of these studies have also demonstrated superiority of echinocandin therapy over triazoles in terms of clinical success and/or microbiological clearance. A retrospective substudy of one randomised trial did suggest that increased cidality may explain these beneficial effects of anidulafungin relative to fluconazole (Reboli et al. 2011).

Our study does have significant limitations. One relates to the question of attributable mortality. Therapeutic success rates were higher among patients treated with fungicidal therapy when compared to fungistatic therapy. Despite this trend however, overall mortality rates in both groups were similar. Although we believe this divergence in therapeutic success and mortality is most likely due to late renal toxicity associated with amphotericin B therapy, an alternate explanation for the failure to translate improved therapeutic success to improved mortality is the possibility of an absence of attributable mortality due to candidemia among non-neutropenic hosts. While available data suggests a significant attributable mortality with candidemia (Falagas et al. 2006) (and presumably, other forms of invasive candidiasis), our analysis is unable to definitively differentiate between the two possibilities.

In addition, salvage therapy administered to patients who, in the opinion of the primary physicians, were not responding to the initial therapy may also differentially introduce a survival bias in triazole therapy group since they had a greater incidence of therapeutic failure. This could attenuate any difference in survival that might otherwise be seen in the two groups.

Another limitation to our analysis is our inability to parse the impact of time to appropriate antimicrobial therapy and time to source control, particularly central venous catheter removal, on outcome. Studies have shown that outcome of *Candida* sepsis is closely related to both factors (Garey et al. 2006; Kumar et al. 2006; Kollef et al. 2012). Although several studies in our data set did comment on the frequency of catheter removal, outcomes were not stratified by this variable (Phillips et al. 1997; Rex et al. 2003; Kullberg et al. 2005). None of the studies reported on time to appropriate antimicrobial therapy. Both represent possible confounders of mortality that are not taken into account in this meta-analysis.

A necessary element of our analysis is that the fungicidal group frequently involved combination therapy. In two cases (Kujath et al. 1993; Abele-Horn et al. 1996), amphotericin B therapy was combined with flucytosine, another fungicidal agent thought to have indifferent or additive effects in amphotericin B treatment of serious fungal infections (Lewis and Kontoyiannis 2001). In three other cases, a triazole was added to amphotericin B during (Rex et al. 2003) and/or following (Kullberg et al. 2005; Reboli et al. 2007) amphotericin B treatment. Most analyses of in vivo data similarly tend to suggest indifferent or additive effects with this combination (Sugar 1995; Sugar and Liu 1998). We believe available data justify the classification of combination therapy in these cases as fungicidal in nature.

Our meta-analysis of candidemia trials evaluating the treatment efficacy between fungicidal and fungistatic therapies among non-neutropenic groups of patients is in concordance with published expert panel guidelines on the treatment of candidemia. The guidelines suggest the use of a fungicidal strategy (echinocandins) rather than fungistatic therapy (azoles) for the treatment of severe infections due to candidemia (Bow et al. 2010; Pappas et al. 2015). Echinocandins offer excellent fungicidal activity without the marked nephrotoxicity and infusion-related side effects associated with amphotericin B as well as offer improved spectrum of activity against azole-resistant *Candida* species.

These data may also have import more broadly with respect to life-threatening infections caused by other pathogens. Although a consensus favours lack of impact of cidal therapy on most infections (Nemeth et al. 2015), there is contrary data with respect to severe infections (Lepper and Dowling 1951; Prasad et al. 2012).

The tenets of septic shock therapy, namely source control, early and appropriate antimicrobial therapy, and utilisation of microbicidal drug strategies when possible in severe infections, should be translated to the treatment of severely ill patients with candidemia and invasive candidiasis.
